# Targeting the Glucocorticoid Receptors During Alcohol Withdrawal to Reduce Protracted Neurocognitive Disorders

**DOI:** 10.3389/fpsyt.2019.00580

**Published:** 2019-09-18

**Authors:** Daniel Béracochéa, Nicole Mons, Vincent David

**Affiliations:** ^1^Université de Bordeaux, Institut de Neurosciences Cognitives et Intégratives d’Aquitaine, Pessac, France; ^2^CNRS UMR 5287, Institut de Neurosciences Cognitives et Intégratives d’Aquitaine, Pessac, France

**Keywords:** alcohol withdrawal and relapse, benzodiazepines, baclofen, corticosterone, gaba receptors, glucocorticoids, prefrontal cortex, working memory

## Abstract

Persistent regional glucocorticoid (GC) dysregulation in alcohol-withdrawn subjects emerges as a key factor responsible for protracted molecular and neural alterations associated with long-term cognitive dysfunction. Regional brain concentrations of corticosterone vary independently from plasma concentrations in alcohol-withdrawn subjects, which may account for the treatment of alcohol withdrawal–induced persistent pathology. Thus, from a pharmacological point of view, a main issue remains to determine the relative efficacy of compounds targeting the GC receptors to attenuate or suppress the long-lasting persistence of brain regional GC dysfunctions in abstinent alcoholics, as well as persistent changes of neural plasticity. Data from animal research show that acting directly on GC receptors during the withdrawal period, *via* selective antagonists, can significantly counteract the development and persistence of cognitive and neural plasticity disorders during protracted abstinence. A critical remaining issue is to better assess the relative long-term efficacy of GC antagonists and other compounds targeting the corticotropic axis activity such as gamma-aminobutyric acid A (GABA_A_) and GABA_B_ agonists. Indeed, benzodiazepines (acting indirectly on GABA_A_ receptors) and baclofen (agonist of the GABA_B_ receptor) are the compounds most widely used to reduce alcohol dependence. Clinical and preclinical data suggest that baclofen exerts an effective and more powerful counteracting action on such persistent cognitive and endocrine dysfunctions as compared to diazepam, even though its potential negative effects on memory processes, particularly at high doses, should be better taken into account.

## Introduction

Alcoholism is characterized by periods of sustained alcohol consumption, in part due to changes in neural circuits mediating anxiety and stress disorders, notably the prefrontal cortex (PFC) and structures such as the hippocampus (HPC) and the amygdala (AMG) (1, 2). Indeed, the PFC–HPC–AMG circuit plays key roles in modulating neuroadaptive responses to stress and anxiety and is markedly and consistently altered in most of neuropsychiatric disorders (3, 4).

Alterations of hypothalamic–pituitary–adrenal (HPA) axis activity is a prime mechanism contributing to protracted alcoholism (5) and the release of glucocorticoids (GCs; cortisol in humans and primates, corticosterone in rodents) from the adrenal glands. Clinical and experimental data in both humans (6–8) and rodents (5, 9, 10) have shown that both acute and chronic alcohol consumption, as well as alcohol withdrawal, enhanced plasma GCs and decreased GC receptor (GR) availability (11). In addition, even though the relationships between HPA axis activity, craving, and alcohol intake during early abstinence have been particularly well documented (4, 12), little is known on such a relationships during protracted abstinence. Moreover, whereas most of the measures of the HPA axis activity are peripheral, some brain regions playing a critical role in either memory or reward processes have been shown to exhibit sustained local GC dysfunction in contrast to a transient increase in circulating GC level, a phenomenon that is as yet insufficiently taken into account to understand alcohol relapse in abstinent subjects (13, 14).

In the first part, this review thus provides updated clinical and experimental evidence for the persistence of brain regional GCs over protracted alcohol abstinence and how sustained GC-related neurocognitive dysfunction might possibly lead to relapse. In the second part, this paper focuses on the efficacy of pharmacological compounds modulating, directly or indirectly, GC receptors to suppress or attenuate these long-lasting neurocognitive alterations in alcohol-withdrawn subjects. Even though alcohol withdrawal affects numerous brain structures and networks (15–17), in this review, we focused on PFC-related studies. Indeed, on the one hand, it has been shown that neurons of the PFC are dramatically vulnerable to the oxidative stress mediated by chronic alcohol exposure, leading to important neuronal cell death (18), and on the other hand, our own studies have shown that alcohol withdrawal induced protracted GC alterations in the PFC that were responsible for working memory (WM) impairments in mice (13, 19, 20).

## Persistent Brain Regional Glucocorticoid Alterations After Protracted Alcohol Abstinence

Alcohol withdrawal induced protracted alterations of corticosteroid-releasing factor (CRF) and plasma corticosterone in the HPC, the PFC, and the hypothalamus, far beyond the detoxification step (10). Interestingly, the long-lasting neuroadaptive changes of GCs caused by prolonged alcohol withdrawal within neural circuits involved in learning, memory, and emotions are only scarcely known.

**Figure 1:**
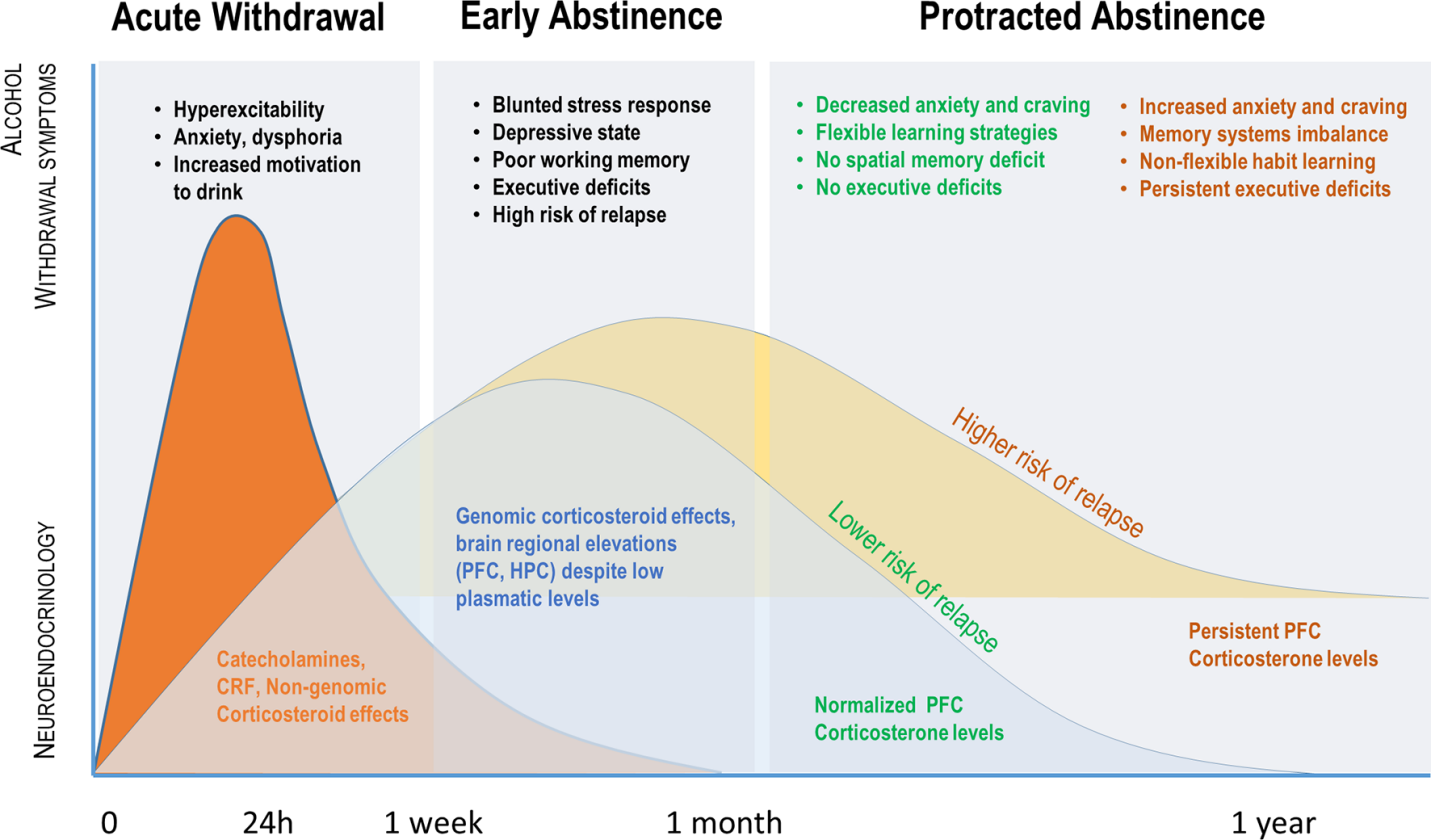
Parallel evolution of alcohol withdrawal–induced symptoms and plasmatic or brain regional glucocorticoid (GC) levels over time. Acute withdrawal is associated with a release of catecholamines, CRF, and high plasmatic GCs, which mediate physiological and behavioral symptoms initially through non-genomic effects. The early abstinence period is associated with a decrease in plasmatic GC concentration as opposed to a brain regional GC increase, particularly in the PFC, likely involving genomic effects of GC. Although brain GC concentration and affective/cognitive symptoms will be normalized in many dependent subjects, persistence of elevated brain GC levels and cognitive impairments in others is predictive of high risk of relapse (see text for details).

The initial phase of alcohol withdrawal is characterized by increases of both plasma and brain GC concentrations (13, 14, 19, 21). Little and colleagues (14) were the first to show in rodents that during the initial phase of withdrawal after 8 months of chronic alcohol consumption (CAC), rats and mice showed exaggerated corticosterone levels in the PFC and the HPC. The excessive corticosterone level in the PFC of alcohol-withdrawn rodents persisted for up to 2 months, whereas circulating corticosterone level already returned to basal concentrations. Other studies also reported that protracted high levels of local corticosterone concentration are important factors for the maintenance of cognitive impairments after prolonged cessation of alcohol intake in rodents (19, 22, 23) and in abstinent patients (11, 24). The persistence of altered regional GC responses to long-term alcohol withdrawal could be a clue to understand how the local neuroadaptive changes to withdrawal generate sustained downstream molecular and neurofunctional activity disorders, notably in the PFC–HPC–AMG circuit, and could promote relapse to alcohol-seeking behavior (see **Figure 1**).

In our own studies, *in vivo* intracerebral microdialysis was used to evaluate the evolution of corticosterone concentration over time in the medial PFC and the dorsal HPC during and after completion of a WM task in mice previously submitted to a 6-month CAC period followed by either a short (1 week) or prolonged (6 weeks) withdrawal period (13, 19). This WM task was based on spontaneous alternation behavior, which involves intact interconnections between the PFC and the HPC for successful performance (25, 26). We observed that behavioral testing produced an exaggerated corticosterone rise in the medial PFC regardless of withdrawal duration, in spite of normal circulating GC levels. In addition, a late deficit in the inhibitory feedback response on HPA axis activity in both the PFC and the dorsal hippocampus (dHPC) was also observed in withdrawn mice, even though unrelated with the WM deficit (19). Interestingly, the severity of the memory deficit correlated positively with high levels of PFC corticosterone concentration, showing that there is a functional link between exaggerated corticosteroid responses and PFC-related cognitive dysfunction (27–29). The persistent elevation in PFC corticosterone levels in withdrawn mice could be due to the presence of local GC production, proximity to target cells, and possibly, tissue-specific control mechanisms (30). Our findings agree with many neuroimaging studies showing structural and functional deficits in PFC regulatory regions (31) or blood flow alterations in the medial frontal lobe (32). Thus, a functional disconnection between the PFC and the AMG emerges as an early index of neuroadaptation in alcohol dependence that predicts PFC-dependent cognitive impairments during abstinence (29, 33, 34). Endogenous GCs are critically implicated in maintaining PFC-dependent cognitive functions (35). Consistently, an increase in cortisol predicts frontal cortex–related cognitive deficit as shown either after a hydrocortisone administration or in pathological condition such as in Cushing’s disease (33, 36–39).

## Regional GC Alterations and Neural Plasticity

GCs influence brain function through two types of GRs, the high-affinity mineralocorticoid receptor (MR) or the low-affinity GR (40). GR acts as a nuclear transcription factor to regulate expression of various target genes (41–43). They also play an important role in the addiction to alcohol. For instance, GR-mediated plasticity increased voluntary alcohol intake (44), whereas GR antagonist reduced alcohol intake (45) in rats. Thus, alterations of GCs in withdrawn subjects could impair neural plasticity within the PFC–HPC–AMG circuitry implicated in stress and higher cognitive function, such as declarative memory and WM (19, 23, 29, 46, 47).

Several lines of research have shown that GC alterations disrupt memory processes through either changes in activated/phosphorylated cAMP response element-binding protein (pCREB) due to GR dysfunction (48–50) or the MR-mediated effects on the cAMP–protein kinase A (PKA) cascade (51, 52). A recent study in our lab reported that early and protracted withdrawal periods after prolonged alcohol consumption produced WM deficits in a sequential alternation task, which were associated with reduced pCREB levels, more specifically in the PFC, whereas none of these impairments were observed in mice still under alcohol condition (13). Results further indicated that local injection of the PKA activator (Sp-cAMPS) into the PFC significantly improves or impairs, respectively, WM performance in withdrawn and water animals (13). These findings strongly support the view that dysregulation of the cAMP–PKA–CREB signaling pathway, particularly in PFC, is a key molecular signature of the cognitive dysfunction during alcohol withdrawal (53–56). The impact of GCs on PFC function is thought to be driven mainly, although not exclusively (57), *via* complex local interactions between dopaminergic and glutamatergic receptors (58, 59).

## Rescuing Long-Lasting Withdrawal-Induced Cognitive and Glucocorticoid Dysfunction by Targeting GC Receptors

The highest densities of MR are expressed in the HPC (60–62). In contrast, the GRs are widely distributed throughout the brain (40, 63, 64), mainly in areas involved in learning and memory. These areas are particularly sensitive to the effects of stress, namely the PFC–HPC–AMC.

AMG circuitry (65–67). More specifically, as shown in human studies of Cushing’s syndrome, sustained cortisol elevation over the years alters the integrity of the HPC–PFC circuitry and accordingly influences the severity of various cognitive dysfunctions (37, 38, 68–70). Indeed, GC impairment of WM critically depends on influences within the PFC (27–29), and selective impairments of frontal cortical functions during withdrawal in detoxified alcoholics have been reported (16, 34, 71–74). These findings are in agreement with several studies indicating that exaggerated concentrations of GCs produced PFC dysfunction, as also reported in depression or Cushing’s syndrome (16, 36, 37, 68, 75–78).

Several types of pharmacological compounds acting on GC release or the GC receptors have been used to restore memory function after alcohol withdrawal. Thus, mifepristone (a GR antagonist) or the dihydropyridine calcium channel nimodipine, delivered prior to withdrawal from chronic alcohol exposure, reduced both the protracted rises in brain corticosterone and sustained cognitive or motivational deficits in mice (22) or rats (79). Recently, we studied whether the regional GC blockade in the medial PFC suppressed WM deficits in alcohol-withdrawn mice. To that aim, withdrawn mice were given intraperitoneal administration of metyrapone (a corticosterone synthesis inhibitor) prior to testing. We found that the withdrawal-induced WM impairments were totally alleviated, confirming the key role of persistent enhanced GC levels in withdrawal-associated cognitive impairments. Similarly, acute intra-PFC infusion of spironolactone that diminished MR activation, and to a lesser extent, of mifepristone that diminished GRs activation, fully restored WM function in withdrawn mice. In contrast, neither spironolactone nor mifepristone had any effect when infused into the dorsal HPC (19). These data are congruent with findings reporting that high GC levels *via* either corticosterone administration or local infusion of the GR agonist RU 28362 into the medial PFC shortly before testing similarly impair WM (29), while the GR antagonist RU 38486 infused into the PFC can restore stress-induced deficits in executive function (59). All together, these findings suggest that long-term adaptive behavioral effects of withdrawal after a long alcohol exposure are mediated in large part through sustained GC dysregulation within the PFC circuitry, while circulating corticosterone levels are already normalized.

## Recovery of PFC Functions and Successful Protracted Abstinence

There is now extensive evidence showing that recovery of PFC cognitive function is related to long-lasting abstinence in alcoholics (16, 80, 81). This raises two critical issues that should be addressed by future clinical and animal research. Firstly: is recovery of executive functions relying on restoration of normal PFC activity, or is it the result of compensatory activity in other cortical or hippocampal regions as previously suggested (82)? For instance, it remains unclear whether some of the withdrawal-induced cognitive impairments are due to the PFC itself or the HPC (16, 73, 74, 83, 84). Since many confounding factors may limit the relevance of clinical studies in that matter, it is an essential task of preclinical models of alcohol dependence to better understand regional cellular substrates of these cognitive deficits. Secondly, GC release corresponds to a physiological mechanism (negative feedback control), which is preparing the organism to cope and eventually to recover from various environmental threats (85). The medial PFC is a critical target area for the negative-feedback effects of GCs on HPA activity after stress (86). There is increasing evidence showing that PFC-dependent cognitive impairments in many alcohol-dependent subjects are no longer observed after 1 year of abstinence (81, 82, 87). Therefore, a fundamental issue will be to better evaluate the long-term benefits of targeting GC activity, in order to determine what GC-related treatments are effective in reducing transient withdrawal-induced cognitive deficits without compromising normalization of the stress system reactivity and cognitive function.

## Targeting GC Activity During Withdrawal *via* GABAergic Agonists

Another way to rescue the protracted regional GC dysregulation in alcohol-withdrawn subjects and rodents is to act directly or indirectly on the GABAergic neurotransmission during the withdrawal period. Indeed, the GABAergic system modulates the HPA axis response to stress (88–91) mainly through its inhibitory action on corticotropin-releasing hormone (CRH) cells of the paraventricular nucleus of the hypothalamus, which regulates GC release by the adrenal gland (92). Experimental data have already shown benefic effects of GABA_A_ agonist (muscimol) on alcohol tolerance and dependence in rats (93). However, among benzodiazepines (such as lorazepam, chlordiazepoxide, and oxazepam) acting on the GABA**_A_** receptor, diazepam is the most commonly used, mainly given its prolonged half-life (94). Given that, diazepam has been widely used to reduce the negative side effects of alcohol withdrawal and transiently delivered in alcoholics mainly with the aims of reducing anxiety and decreasing neural excitability in the early phase of the cessation of alcohol intake (95–100).

However, given the high variability of patients’ reactions to diazepam, its use may also be causal of strong deleterious neurocognitive and affective disorders (101). For example, diazepam induces deleterious effects on cognitive functions (mainly amnesia) in humans (102–104) and rodents (89, 105, 106) that resemble those induced by chronic alcohol consumption and withdrawal (105). In addition, it is well established that addiction to benzodiazepines can develop over time in treated alcoholics or in people with a history of a substance use disorder (100, 107). Furthermore, chronic diazepam treatment potentiates the addictive properties of psychostimulants such as cocaine (108). In a rodent model of chronic intermittent access to alcohol leading to escalation of alcohol intake, George et al. (109) showed that recruitment of GABAergic and CRH cells in the medial PFC during withdrawal and disruption of the PFC–central AMG pathway are causal factors for impairments of executive control over motivated behavior, suggesting that alterations of medial PFC interneurons may be a prime signature of neuroadaptation in dependence on alcohol. Interestingly, functional inactivation of the orbitofrontal cortex by agonists of the GABA_A_ (muscimol) and GABA_B_ (baclofen) receptors disrupts the context-induced relapse to alcohol and executive control in rats (110). Overall, in spite of motivational and cognitive disorders potentially linked to the use of benzodiazepines, they remain the most common pharmacological compounds used to reduce the negative side effects of alcohol withdrawal in humans. Indeed, other compounds such as anticonvulsant drugs (carbamazepine, valproic acid, or gabapentin, for instance) and barbiturates such as phenobarbitone also attenuate alcohol withdrawal symptoms, but their use is often limited by negative side effects or insufficient benefic effects as compared to benzodiazepines [for a comprehensive review, see Ref. (111)].

**Figure 2:**
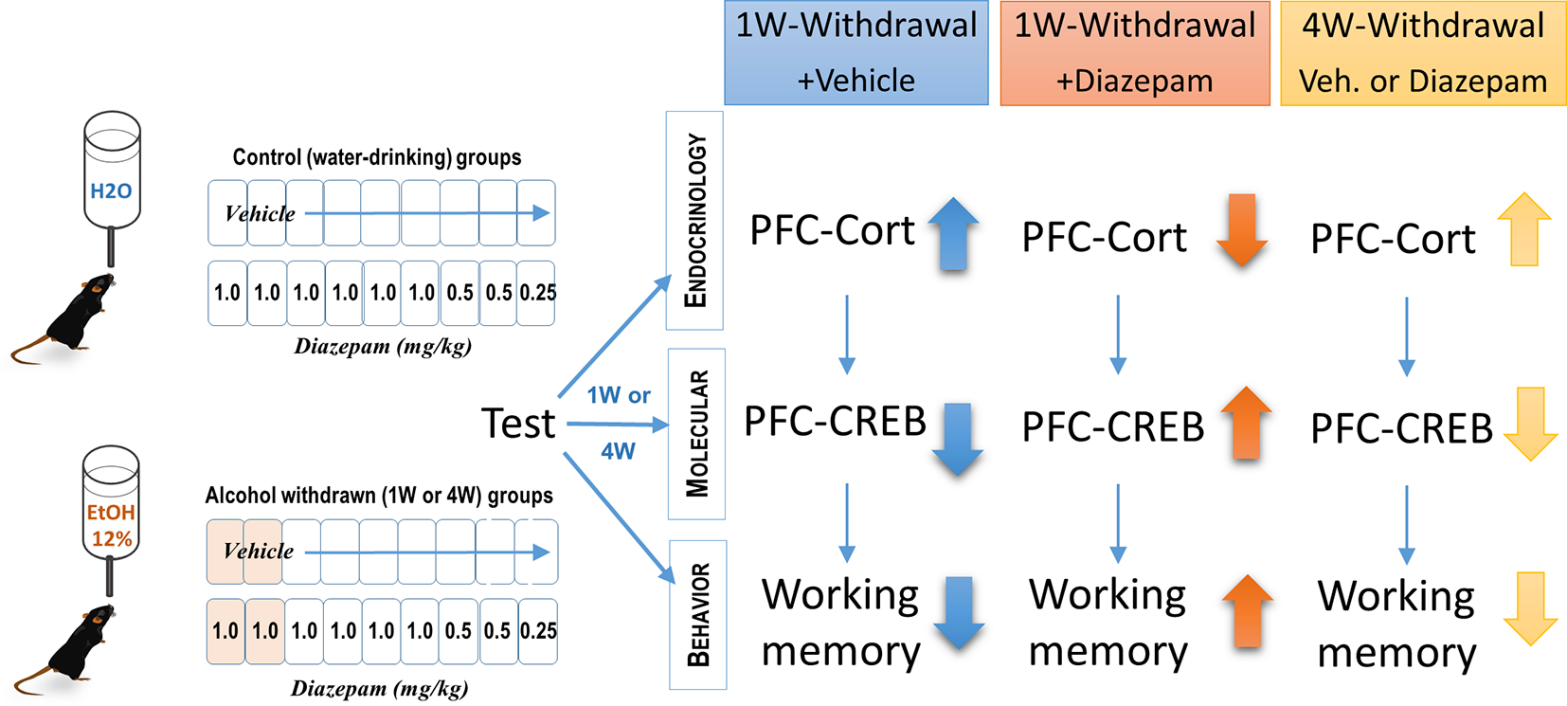
Effects of diazepam treatment on cognitive deficits, prefrontal cortical (PFC) GC levels, and pCREB expression in alcohol-withdrawn C57BL6/J mice. Chronic alcohol consumption lasted 6 months at 12% (v/v). This pharmacological study showed that 1-week-withdrawn mice receiving vehicle exhibited increased levels of corticosterone, reduced pCREB activity in the PFC, and working memory deficits as assessed with a sequential alternation task, 24 h after the last diazepam injection. Diazepam administered i.p. at decreasing doses ranging from 1.0 to 0.25 mg/kg every day during the 9 days of the withdrawal phase transiently (1 week but not 4 weeks) reversed both the endocrine and cognitive impairments observed in vehicle-treated animals (128).

Initially used for its myorelaxant effect through its agonist action on GABA_B_ receptors (112), baclofen was found to modulate HPA axis activity (113) and to reduce HPA axis activity in withdrawn alcoholics (114). Baclofen has been used only recently in the treatment of alcohol dependence (97, 115, 116). Although the initial case report put an emphasis on its anticraving properties (117), increasing evidence suggests that different mechanisms could account for the effects of baclofen on motivational and physical symptoms of alcohol withdrawal (115–119). Clinical and experimental data have mentioned an attenuation of alcohol dependence in both humans and animals, even though adverse events have been also reported, mainly with high baclofen doses (110, 120–123). Whereas beneficial effects of both diazepam and baclofen after a short period of alcohol withdrawal are well documented, a critical issue that remains under question is to determine the relative efficacy of these compounds at rescue from the persistent cognitive and biological alterations resulting from long withdrawal periods. In humans, diazepam and baclofen induced comparable and similar physical symptoms to those of alcohol withdrawal, such as anxiety, sweating, and tremors over a 10-day withdrawal period (124). A recent survey study did not report different qualitative effects of baclofen as regards other benzodiazepines (diazepam, chlordiazepoxide) in the treatment of severe alcohol withdrawal syndrome (125). In contrast, another study showed a greater efficacy of chlordiazepoxide as compared to baclofen in reducing the physical symptoms of alcohol withdrawal (126). Low doses of baclofen associated with benzodiazepine administration lowered the dose of lorazepam used to counteract the increase in anxiety resulting from the cessation of alcohol intake (127). We recently confirmed the corrective effects of a 9-day diazepam administration on memory dysfunction, GC levels, and altered pCREB in the PFC after a short (1 week) withdrawal period in mice; however, these benefic effects were only transient since they were not observed after a longer (4 weeks) alcohol withdrawal period (128) (and see **Figure 2**). The lack of efficacy of subchronic diazepam injections to alleviate the protracted cognitive and biological alterations in 4-weeks-withdrawn mice may result from sustained alterations of GABA_A_ receptors (99, 129, 130), increased downregulation of these receptors over repeated diazepam administration (131), or other neuroadaptations that may progressively emerge after withdrawal, such as alterations of epigenetic mechanisms (4, 20).

In contrast to diazepam, other studies have reported beneficial effects of baclofen after protracted alcohol withdrawal. More specifically, Geisel et al. (114) evidenced in abstinent alcoholics sustained increased plasma GC levels, which decreased significantly in baclofen-treated patients, up to 14 weeks after treatment. Authors suggested that a decrease of CG levels during treatment with high-dose baclofen contributes to its preventive effects on alcohol relapse. In line with this hypothesis, we reported in recent experiments, as yet unpublished, a clear-cut dissociation between baclofen and diazepam in the protracted GCs and motivational dysfunction in alcohol-withdrawn mice. Using an odor place preference paradigm, we showed that alcohol-withdrawn animals receiving an acute stress (electric foot shocks) before the recognition session exhibited an abnormal rise of plasma corticosterone as compared to stressed controls, as well as a strong preference for an area impregnated with the odor of alcohol at the expense of a zone impregnated with water. Interestingly, repeated administration of baclofen administered during the withdrawal period normalized the stress-induced plasma corticosterone rise and concomitantly suppressed the stress-induced alcohol place preference, up to 4 weeks after the cessation of alcohol intake, whereas diazepam had only a short transitory (1 week) beneficial effect (132).

In spite of its promising effect in the treatment of protracted alcohol-related neurocognitive and motivational disorders, one might note that a limitation of the use of baclofen is associated with the determination of the relevant dose to induce beneficial effects without negative side disorders (116) such as cognitive and emotional disorders, which have been reported with high doses (133). Its use in humans might require closet medical surveillance given the pathological alterations associated with self-misuse or high doses of this compound.

## Concluding Remarks

From a functional point of view, persistent regional GC dysregulation in alcohol-withdrawn subjects emerges as a key factor responsible for protracted molecular and neural alterations associated with long-term cognitive dysfunction. The demonstration that regional brain concentrations of GCs can change in alcohol-withdrawn subjects independently from circulating concentrations has important implications for the treatment of alcohol withdrawal–induced persistent pathology. Thus, from a pharmacological point of view, a main issue remaining to be resolved concerns the relative efficacy of compounds targeting the GC receptors to attenuate or suppress the long-lasting persistence of brain regional GC dysfunctions in abstinent alcoholics, as well as other persistent changes of neural plasticity. Data from animal experimentation show that acting directly on GRs during the withdrawal period, *via* selective antagonists, can significantly counteract the development and persistence of cognitive and neural plasticity disorders during protracted abstinence. A critical remaining issue is to better assess the relative long-term efficacy of GABA_A_ and GABA_B_ agonists in counteracting the protracted brain regional GCs and neurocognitive dysfunctions resulting from alcohol withdrawal. Clinical and preclinical data suggest that the agonist of the GABA_B_ receptor baclofen exerts an effective counteracting action on such persistent dysfunctions. However, there is still a need for a better evaluation of its potential negative side effects, particularly when using high doses over a long period of time.

## Author Contributions

DB and VD wrote the paper. NM edited it.

## Funding

This work was supported by the Centre National de la Recherche Scientifique (CNRS), The University of Bordeaux, and the Regional Council of the Aquitaine Region (DB, VD).

## Conflict of Interest Statement

The authors declare that the research was conducted in the absence of any commercial or financial relationships that could be construed as a potential conflict of interest.
